# Online Survey for the Assessment of Generic Health Literacy among Adolescents in Germany (GeKoJu): Study Protocol

**DOI:** 10.3390/ijerph17051518

**Published:** 2020-02-27

**Authors:** Anne-Kathrin M. Loer, Olga Maria Domanska, Ronny Kuhnert, Robin Houben, Stefan Albrecht, Susanne Jordan

**Affiliations:** Department of Epidemiology and Health Monitoring, Robert Koch Institute, General-Pape-Str. 62-66, 12101 Berlin, Germany; DomanskaO@rki.de (O.M.D.); KuhnertR@rki.de (R.K.); HoubenR@rki.de (R.H.); AlbrechtS@rki.de (S.A.); JordanS@rki.de (S.J.)

**Keywords:** health literacy, youths, measurement, cross-sectional study, representativeness

## Abstract

The promotion of health literacy at a young age can protect, maintain and improve health across the life course. Yet to date, a sound data basis on adolescent health literacy as a requirement for the development of strategies to promote health literacy has not been given. This paper presents a study protocol for the online survey “Health Literacy Among Adolescents” (GeKoJu) that collects the first nation-wide representative data on self-reported generic health among adolescents aged 14–17 years in Germany. The objectives of the survey are (1) to assess the distribution of generic health literacy among adolescents in Germany, (2) to identify socio-demographic and social factors in regard to health literacy and (3) to assess the association of health literacy and health-related outcomes. The cross-sectional survey was conducted from September 2019 through December 2019. A two-stage stratified cluster sampling strategy was applied. Individuals invited to participate in the survey (*N* = 6608) were randomly selected among German-speaking adolescents aged 14–17 years, with permanent residence in Germany. Generic health literacy is measured with the “Measurement of Health Literacy Among Adolescents-Questionnaire” (MOHLAA-Q). Data collection also covers questions on health behavior, subjective health status, personal and social resources, socio-demographic and social factors and health services use. Results of the GeKoJu survey will provide data for the development of strategies to promote generic health literacy among families, in schools, communities and health care.

## 1. Introduction

### 1.1. Background

Health literacy is attracting increasing attention in the public health field worldwide [1,2]. According to the widespread definition by Sørensen et al. [2] (p. 3), “*Health literacy is linked to literacy and entails people’s knowledge, motivation and competences to access, understand, appraise, and apply health information in order to make judgments and take decisions in everyday life concerning healthcare, disease prevention and health promotion to maintain or improve quality of life during the life course*”. Previous studies have mostly examined health literacy among adults. These studies showed that a low health literacy level is associated with perceived difficulties in accessing, understanding, evaluating and applying health information [3]. Further study results point out that a lower health literacy level is associated with a riskier health behavior, a poorer general health status, higher morbidity and mortality and higher total expenditure in health systems [3,4,5,6,7,8,9]. Health literacy is also discussed being associated with various antecedents of health disparities [10]. Therefore, strengthening health literacy has been receiving increased attention for reducing social inequalities [4,9,11,12].

The promotion of health literacy at a young age can have positive health effects across the life course [13,14,15,16]. As a result, the World Health Organization has identified children and adolescents as a relevant target group for the promotion of health literacy. This target group is characterized by specific vulnerabilities e.g., to risky behavior, such as substance misuse [17], or to demographic factors. These vulnerabilities are relevant for health literacy as they lead to specific health information and resource needs [14]. However, for promoting health literacy in this target group, a sound data basis for adolescents’ perceived relevance as well as the meaningfulness of health information and their current health literacy level and its distribution is a necessary condition for the development of any evidence-based interventions [14,18]. To date, less attention has been paid to the collection of dates focusing on adolescent health literacy [19,20,21,22], a situation which also applies to Germany. Few studies measuring adolescent health literacy focus on specific health literacy domains, age groups or subgroups [23,24,25,26,27,28,29]. There is no evidence concerning health literacy among 14−17 year-olds and only one population-based study drew a sample from population registries [27]. Until now, there have been no nation-wide representative data on self-reported generic health literacy focusing on adolescents in Germany. To close this gap, the online survey on “Health Literacy Among Adolescents” (GeKoJu) is being conducted in Germany. The online survey GeKoJu is part of the project ”Measurement of Health Literacy Among Adolescents”—Part Two (MOHLAA 2), carried out by the Robert Koch Institute (RKI). The RKI is the government’s central scientific institution in the field of biomedicine in Germany. It is charged with identification, surveillance and prevention of diseases as well as monitoring and analyzing long-term public health trends in Germany. The MOHLAA 2 project is part of the German Health Literacy in Childhood and Adolescence (HLCA) Consortium, funded by the German Federal Ministry of Education and Research (BMBF—grant number 01EL1824D) [20].

### 1.2. Objectives

MOHLAA 2 aims to provide the first nation-wide representative data on generic health literacy among adolescents in Germany. The GeKoJu survey objectives include: Objective 1: to assess the distribution of generic health literacy among adolescents in Germany; Objective 2: to identify socio-demographic and social factors related to health literacy; Objective 3: to assess the association of health literacy and health-related outcomes (e.g., subjectively assessed health status and health behavior). The following protocol presents the research design and methods as well as a discussion of methodological challenges, strengths and limitations relating to the study design and sampling strategy. The findings of the study will be presented in later publications and not in this publication, since the format of a study protocol does not aim to provide a presentation of results and conclusions. This approach allows the scientific community to assess whether collected data and prospective results are consistent with the original intent of the study [30]. The study protocol with essential information about the execution of our survey is drawn up to inform other international researchers focusing on health literacy and adolescents, as well as support them in their own study planning [30,31].

## 2. Research Design, Methodology, and Methods

### 2.1. Study Design

The study is a cross-sectional study measuring generic health literacy among adolescents living in Germany. Due to the fact that individuals from the target population use the internet at least several times per week, if not daily [32], data were collected online (Computer Assisted Web Interview-CAWI). Survey planning was carried out under the direction of MOHLAA 2 researchers within the “Health Behavior” unit at the RKI. To ensure data protection, the study design ensured there was separate collection, processing and storage of personal data and survey data. Personal data and survey data are handled by separate units at the RKI, also in cooperation with an external contractor, the market and social research institute USUMA GmbH.

### 2.2. Study Population

Individuals were selected for study participation according to the following inclusion criteria: (1) German-speaking adolescent with permanent residence in Germany, (2) aged 14–17 years at the date of sampling, (3) randomly selected from data in resident registration offices and (4) who provided their written informed consent form, with a personal and parent/guardian signature.

### 2.3. Sampling Strategies

The distribution of sample points is precisely planned and includes all regions in Germany, to achieve a nationally representative sample. The sampling protocol was developed in cooperation with GESIS Leibniz Institute for the Social Sciences, Mannheim, Germany. The survey sample was obtained using two-stage stratified cluster sampling.

**Figure 1:**
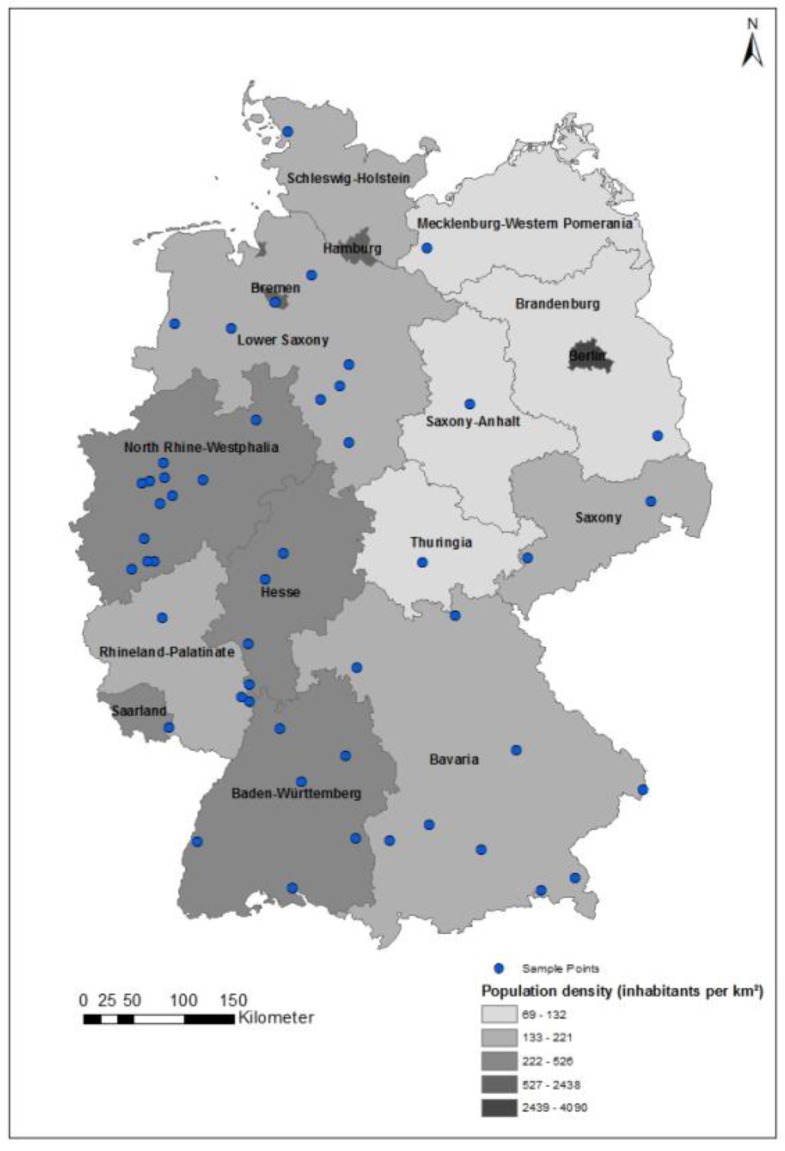
Sample points of the online survey Health Literacy Among Adolescents (GeKoJu) (*n* = 50).

Primary sampling units (PSUs) were sampled from an inventory of German communities stratified by district and according to the “BIK” classification system, which reflects the grade of urbanization, regional population density and administrative borders [33]. At the first stage, 50 sample points were selected throughout 13 federal states (“Bundesländer”) in Germany (see Figure 1).

In the second stage, a pre-defined number of addresses were randomly drawn from local population registers at resident registration offices within selected PSUs. It has been observed in nation-wide health surveys [34] and in the RKI’s long-term study “German Health Interview and Examination Survey for Children and Adolescents” (KiGGS study) [35,36,37], that individuals with a migrant background show lower response rates than individuals without a migrant background. To achieve a sample representative of the population’s proportion of youths with a migrant background, a higher number of addresses were drawn in the 10 PSUs with the highest population density, to increase the likelihood of selecting individuals with a migrant background. This approach is based on evidence which demonstrates a tendency of individuals with a migrant background to live predominantly in larger cities [38]. Finally, a random sample of adolescents per birth cohort was drawn based on calculations which consider the community size and sample points (first birth cohort: 01.09.2001–31.08.2002; second birth cohort: 01.09.2002–31.08.2003; third birth cohort: 01.09.2003–31.08.2004; fourth birth cohort: 01.09.2004–31.08.2005). The calculated crude sample size was *N* = 4983 with an expected response rate of 21.75% based on the pilot study MOHLAA 1 in Berlin [39]. The response rate (17.3%) following the first batch of study invitations (*n* = 500) was lower than expected. To assure a net sample size of *n* = 1084, we expanded the crude sample size to *N* = 6608.

### 2.4. Data Protection and Ethics Approval

The study was approved by the Federal Commissioner for Data Protection and Freedom of Information without concern on July 09, 2019. To ensure data protection, the study design warranted separate collection, processing and storage of personal data and survey data. Personal data and survey data are handled in various subdivisions (subject areas) of the Robert Koch Institute and in cooperation with an external contractor, the market and social research institute USUMA GmbH. When processing social data, USUMA GmbH is contractually obliged to observe technical and organizational measures for data protection and data security [40]. Participants as well as their parents/legal guardians received detailed information materials about the study. Informational materials included a cover letter, a survey flyer with detailed survey information, a data protection declaration. Furthermore, USUMA GmbH provided participants with a hotline number and e-mail address. The written informed consent form has to be signed by adolescent and their parents/legal guardians.

Ethics approval was received from the ethics committee at the Alice Salomon Hochschule Berlin, University of Applied Sciences (Number 06-2019/26) on August 8, 2019.

### 2.5. Data Collection & Data Handling

**Figure 2:**
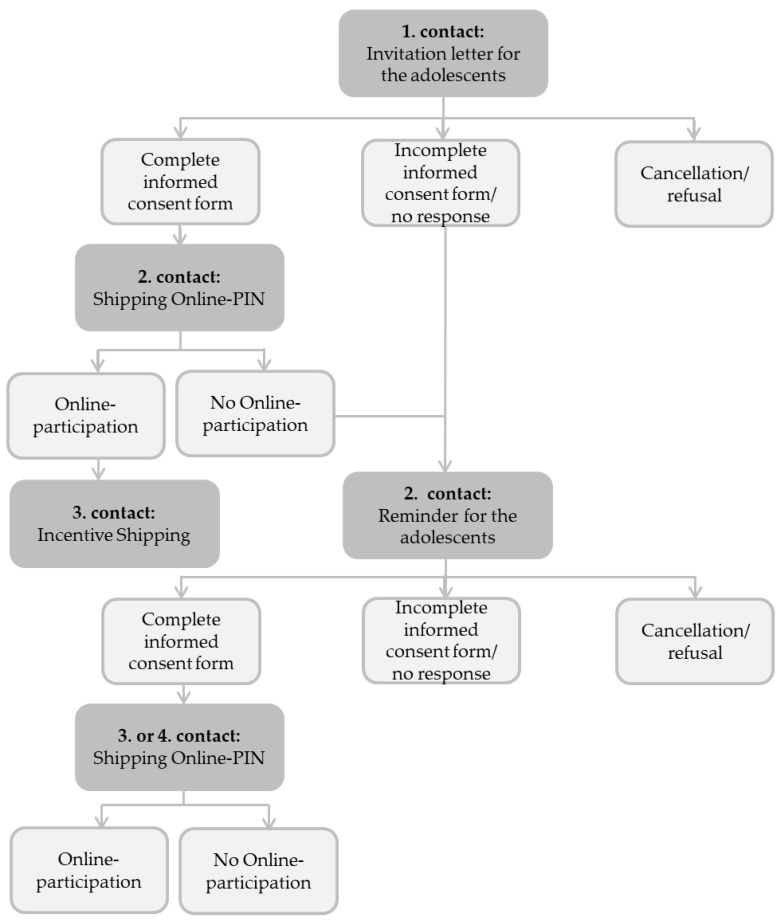
Overview of the study flow and informed consent procedure.

Data are being collected from September until December 2019. The online survey duration is 25 to 30 min. Invitations for participation along with comprehensive informational materials for adolescents and their parents/legal guardians are distributed by USUMA GmbH through the postal mail. After obtaining written informed consent forms, signed by both minors and their parents/legal guardians, a letter containing the personal identification number (PIN) access code is sent to participants. Following the invitation letter, a reminder is sent within three weeks, to potential participants who (1) did not actively decline participation, (2) submitted an incomplete informed consent form or (3) did not participate following receipt of their personal identification access code. Following completion of the online questionnaire, participants received a thank you letter containing a voucher for 8 Euros of credit. Beforehand, they could choose between a voucher for a drugstore or an electronic store. Please see Figure 2 for an overview of the informed consent procedure and study flow.

The online questionnaire was programmed in the online survey and data collection software, VOXCO version 6.0.0.51 (Voxco, Montreal, Canada). The questionnaire was programmed using responsive design to ensure that the display was compatible across a variety of devices (mobile smart phone, notebook, laptop). The team relied on participant verification offered by the VOXCO software to prevent unauthorized participation in the online survey. Participant verification cross-references and verifies the self-reported year of birth and sex using data provided by the resident registration offices. If in some cases the information delivered by the registration office was wrong, then the participants were given the possibility to contact a person responsible via a hotline to adjust the data

### 2.6. Measurements

The standardized online questionnaire contains only scales that have already been validated and applied in other studies. Items capturing socio-demographics, subjective social status and health-related outcomes were assessed in a standardized manner, as previously assessed in the KiGGS study. Unless otherwise stated, the remaining questions were developed from previous population-based surveys at the RKI. Table 1 shows an overview of the topics and instruments used.

The focus of the GeKoJu survey is on generic health literacy as measured by the MOHLAA-Q tool. The instrument was developed and validated in the first funding phase of the project (MOHLAA 1) for use among 14–17 year-olds. The MOHLAA-Q consists of 29 items in four scales (Scale A-D): A. “Dealing with health-related information” (adapted HLS-EU-Q47-GER items, 12 items); B. “Communication and interaction skills” (4 items); C. “Attitudes toward one’s own health and health information” (5 items) and D. “Health-related knowledge” (8 items). The MOHLAA-Q tool is shown in Supplementary Materials. The four scales assess cognitive, behavioral and affective components of generic health literacy. In pretest, the MOHLAA-Q scales yielded acceptable validity indicators. The reliability coefficients (Cronbach’s α) of the scales reported were 0.54 to 0.77 [39]. The psychometric properties of the MOHLAA-Q indicated that the instrument can be used to assess different dimensions of health literacy. The development and validation process of this questionnaire were described elsewhere [39,47].

A further indicator of generic health literacy is an item capturing health literacy seeking behavior (i.e., Please indicate how much health information you have received from the following sources: your parents, health classes in school etc.), adapted from the USA survey “Teens, Health and Technology” [49]. The GeKoJu survey includes 3 items of the Health Literacy Measure for Adolescents (HELMA), which measures reading comprehension and numeracy skills as a proxy for functional health literacy. HELMA Items: 1. If a person drinks 3 cups of milk in one given day, how many carbohydrates has she/he received? (based on the given information in questionnaire). 2. Calculate the BMI of a person with height = 160 cm and weight = 70 kg? 3. What is this person’s BMI status? (based on the given information in questionnaire). Participants were allowed additional aids, such as a calculator, to respond to item 2.

### 2.7. Data Managament, Data Preparation and Data Analysis

Upon closure of the survey, response values will be calculated by USUMA GmbH according to American Association for Public Opinion Research (AAPOR) Outcome Rate Calculator (Mail/specifically named persons) [58].

A weighting factor will be used to ensure that prevalence estimates are representative of the age and gender distribution within Germany. This weighting also accounts for different participation probabilities and corrects for deviations in the design-weighted net sample from the German population, using German population statistics (as of 31 December 2018). Design weighting accounts for a higher number of addresses drawn from 10 sample points. Adjustment weighting is applied to the weighting factors age, gender and school type to guarantee representativeness of the sample and reduce non-response bias. The additional application of citizenship as a weighting factor is currently being tested.

Data transmission for quantitative analysis will be limited to anonymized survey and survey process data. Before conducting statistical analysis, plausibility and consistency checks will be made alongside exclusion of nonvalid cases. In order to meet basic requirements for statistical analysis, the first analytical step will be generation, recoding and categorization of variables. Before carrying out the test procedures, test-specific prerequisites will be verified. The distribution of specific dimensions of health literacy among adolescents (objective 1) will be assessed using descriptive analyses. Bivariate and multivariate analyses will be conducted to assess socio-demographic and social factors related to health literacy (objective 2) and to assess the association of health literacy and health-related outcomes (objective 3).

Data preparation and statistical analyses are planned from January 2020 onwards and will be performed by using the statistic software STATA ^®^ version 15.1 (StataCorp LLC, Texas, TX, USA).

## 3. Discussion

The online survey GeKoJu will provide the first nation-wide representative data on self-reported generic health literacy for adolescents aged 14–17 years in Germany. The data will provide insights regarding the distribution of generic health literacy among adolescents in Germany. The analyses will also provide evidence regarding the association between health literacy levels and health-related outcomes and social factors. The data collected may also being used for the exploration of the relationship between health literacy and health disparities.

A major strength of the survey is the use of the age-adjusted, validated measurement tool MOHLAA-Q which addresses methodological and data gaps. MOHLAA-Q is methodologically tailored to characteristics of adolescents aged 14–17 years and measures generic health literacy in a sample which is representative of the German adolescent population. The use of objective and subjective measurement tools is currently being discussed. On the one hand, it is critically examined whether self-reporting provides valid data concerning health literacy [21]. On the other hand, the question is raised whether an objective measurement can cause feelings of shame or discomfort due to their abilities in low health literate persons [21]. Okan et al. [21] suggest that subjective (self-reported) and objective (performance-based) measurements should be combined for measurement of health literacy, in order to generate more detailed and profound data and richer results. These results may be used for the development of more problem-centered strategies addressing health literacy weaknesses [21]. MOHLAA-Q takes this debate into account and heeds this recommendation in its combination of performance-based items, measuring health-related knowledge and reading comprehension, with self-reported items.

The GeKoJu survey is important based on the public health relevance of health literacy. McCormack et al. highlight the importance of monitoring health literacy prevalence to inform national public health priorities for population health and to reduce inequalities [59]. Understanding the prevalence of health literacy is a first step for the development of evidence-based interventions for health promotion and health care. GeKoJu answers the call of McCormack et al. by collecting nationally representative health literacy data. Information about health literacy deficits, e.g., reading comprehension difficulties or difficulties in seeking health information, could be used to identify vulnerable groups. Survey results will be disseminated to scientists, practitioners and politicians. It can be assumed that the results will be of interest for those groups, as the health literacy concept has become increasingly attention in Germany in recent years. The increased attention is shown, e.g., in the fact that a National Action Plan Health Literacy has been developed by a group of scientists and practitioners supported by the then Federal Minister of Health, which provides scientifically validated guidelines on how health literacy should be promoted in Germany [60].

With regard to the sampling strategy, strengths and limitations can be discussed. Participants were randomly selected through population register sampling. The study pursues the strategy of collecting representative data on a nation-wide level. Hence, we chose a randomly selected sample over snowball sampling or purposive sampling. This selection is the most valid strategy for obtaining a representative sample, because each person in the population has an equal chance of selection [61]. It is used to reduce a sampling selection bias. The strategy of randomly selection of sample points on the first level and participants from local registries on the second level has some advantages over recruiting in single schools, which was also discussed during the study design planning phase. Firstly, by recruiting adolescents in communities across federal states in Germany, it is intended to reduce cluster effects which could occur when recruiting by school class. Secondly, recruitment via population registers allows inclusion of adolescents in various life situations. Adolescents in Germany aged 14–17 year experience an educational transition from secondary level education to one of two tracks: to a longer educational period (secondary level II) or to labor market entry (e.g., combined with a vocational training) [62]. In Germany, the school type in secondary levels I and II varies across federal states [63]. The selected sampling strategy allows visibility of pupils attending various types of secondary level schools, of school leavers and of adolescents who participate in vocational training. However, this sampling strategy is less effective in regard to achieve specific subgroups, e.g., adolescents with a temporary/illegal residence status or a non-German citizenship. One of the reasons for problems of non-accessibility within these subgroups are quality-neutral failures [64,65] such as a selected person has moved or his/her address is unknown. To overcome this limitation, we invited more adolescents living in large urban areas [38]. Further, an adjustment weight factor will be applied to reduce selection bias corresponding to lower response rates of certain groups.

In the current study phase, further limitations in study design should be addressed. The study must be designed to meet data protection standards outlined by the European General Data Protection Regulation (EU) [40]. After feedback from the RKI’s data protection officer on the planned study design, a written informed consent from adolescents as well as their parents/legal guardians must be obtained. Participants may only proceed to complete the online survey after submitting an informed consent form. Following submission of informed consent forms, they receive their PIN by mail. The extended time required to obtain parental/guardian consent, paired with the burden of obtaining parental consent (especially among 17 year-olds who are almost adults with full legal capacity) may affect negatively participant motivation and increase participation burden [66]. This in turn may result in a lower response rate, as observed by participation rates and nonresponse bias in the first batch [66,67]. Due to financial and time constraints, it is not possible to conduct a non-responder survey, which would provide additional insights into the validity of the results. Information regarding socio-demographic characteristics and health risk factors of non-responders as well as reasons for non-participation might help with estimation of non-responder bias [37].

## 4. Conclusions

The results of the GeKoJu survey will supply data which may identify deficits in generic health literacy among youths, which groups are especially vulnerable to health literacy deficits, and factors which influence health literacy among adolescents. Data from the GeKoJu survey may be used for the development of strategies to promote generic health literacy among families, in schools, communities and health care.

## Figures and Tables

**Table 1:** Overview of the topics and measures applied in the GeKoJu survey.

Topic/Parameter	Measures	Indication of Source	Number of Items	Used for Analyzing Follwing Objectives *
**Socio-demographics**	Year and month of birth	Study KiGGS 2 [37]	1	1, 2, 3
	Sex (stated in the birth certificate)	Study “German Health Update” (GEDA19-EHIS) [41]	1	
	Gender (subjective belonging)	Study GEDA19-EHIS [41]	1	
**Education**	School attendence	Study KiGGS 2 [37]	1	1, 2, 3
	Type of school	Study KiGGS 2, slightly adapted [37]	1	
	Achieved school certificate on the secondary level	Study KiGGS 2 [37]	1	
**Socio-economic status of family**	Social Family Affluance Scale	Study “Health Behavior in School-aged Children” (HBSC) [42]	6	1, 2, 3
	Subjective social status (German version of MacArthur Scale)	Study KiGGS 2 [43,44]	1	
	cultural capital through the number of books in the household	Survey PISA 2015 [45]	1	
**Immigration background**	Respondent’s country of origin	Study KiGGS 2 [37]	1	1, 2, 3
	Mother’s country of origin	Study KiGGS 2 [37]	1	
	Father’s country of origin	Study KiGGS 2 [37]	1	
	Length of stay in Germany	Study KiGGS 2 [37]	1	
**Self-reported language skills**	Native language	Project “Improving Health Monitoring in Migrant Populations” (IMIRA) [46]	1	1
	Self-rated German skills	Project IMIRA [46]	1	
	Self-rated native language skills	Project IMIRA [46]	1	
**Generic health literacy**	Measurement of Health Literacy Among Adolescents- Questionnaire (MOHLAA-Q)	Study MOHLAA [39,47]	29	1, 2, 3
**Reading comprehension and numeracy**	Health Literacy Measure for Adolescents (HELMA)	Measurement HELMA, adapted [48]	3	1, 2, 3
**Health literacy seeking behavior**	Sources and quantity of received health literacy information	Survey “Teens, Health and Technology: a national survey”, adapted [49]	14	1, 2, 3
**Health-related behavior**	Alcohol: harmful and binge drinking (Alcohol Use Disorders Identification Test, AUDIT-C)	Study KiGGS 2 [37,50,51]	4	3
	Smoking tabbaco use	Study KiGGS 2 [37]	2	
	Physical activity (sport)	Study KiGGS 2 [37]	1	
	Keeping safety rules	Study KiGGS 2 [37]	4	
	Nutrition (fruits and vegetables consumption)	Study GEDA 2014/2015 [52,53], slightly adapted	4	
**Social and personal resources**	Self-efficacy scale (Scale of General Self-efficacy)	Study KiGGS 2 [54]	10	2, 3
	Social support (the Multidimensional Scale of Perceived Social Support)	Study HBSC [55,56]	8	
**Health status**	Subjective health status	Study KiGGS 2 [37]	1	3
**Anthropometry**	Self-reported height	Study KiGGS 2 [37]	1	3
	Self-reported weight	Study KiGGS 2 [37]	1	
**Utilisation of health services**	Utilisation of doctor visits in the last 12 months	Survey “The European Health Literacy Survey” (HLS-EU), adapted [57]	1	3
	Utilisation of hospitals in the last 12 months	Study KiGGS [35,36]	1	
	Utilisation of ambulances in the last 12 months	Study KiGGS [35,36]	1	
		**Total number**	**106**	

* Objective 1: to assess the distribution of generic health literacy among adolescents in Germany; Objective 2: to identify socio-demographic and social factors related to health literacy; Objective 3: to assess the association of health literacy and health-related outcomes.

## Supplementary Materials

The following are available online at https://www.mdpi.com/1660-4601/17/5/1518/s1, Supplementary file 1: Measurement Health Literacy Among Adolescents Questionnaire (MOHLAA-Q).
