# Supplementary material for: Online Survey for the Assessment of Generic Health Literacy among Adolescents in Germany (GeKoJu): Study Protocol
Source: Int J Environ Res Public Health. 2020 Feb 27;17(5):1518. doi: 10.3390/ijerph17051518 (PMC7084343; doi:10.3390/ijerph17051518)
Supplement: Supplementary file 1 [file ijerph-17-01518-s001.pdf]

Additional file 1. Measurement Health Literacy Among Adolescents Questionnaire (MOHLAA-Q)

Scale A. Dealing with health-related information

	How easy/difficult is it for you to . . .?	very easy (4)	easy (3)	difficult (2)	very difficult (1)
1.	**…find information about what to do when you feel ill to make yourself get better?**	O	O	O	O
2.	**…understand a medication leaflet?**	O	O	O	O
3.	**…judge whether information about medication in the media is credible?** media: internet, TV, radio, press	O	O	O	O
4.	**...follow the instructions of your doctor or pharmacist?**	O	O	O	O
5.	**...find information about how you can deal with mental problems?** e.g., permanent stress, depression, being bullied, an eating disorder	O	O	O	O
6.	**...understand how you can protect yourself from sexually transmitted diseases?** By sexually transmitted diseases we mean diseases such as HIV/AIDS, chlamydia infection, or herpes	O	O	O	O
7.	**...judge whether you can trust media when they warn you of risks to your health?**	O	O	O	O
8.	**...implement recommendations that protect you during sport, in your leisure time and in traffic?** (e.g., wearing a helmet or knee or arm protectors, observing road traffic regulations)	O	O	O	O
9.	**...find information about healthy behavior such as exercise and nutrition?**	O	O	O	O
10.	**...understand information on food packaging?**	O	O	O	O
11.	**...judge how what you do daily affects your health?** e.g., eating, drinking, exercise, relaxation, body care	O	O	O	O

12.	**...get involved in promoting a healthy environment?** e.g., less noise and less traffic, better air quality, more parks and sports grounds	O	O	O	O

Scale B. Communication and interaction skills

	To what extent do you agree with the following sentences?	strongly agree (4)	somewhat agree (3)	somewhat disagree (2)	strongly disagree (1)
13.	**During my last visit to the doctor, I asked all the questions that interested me**.	O	O	O	O
14.	**I chat with my friends about how one can avoid unhealthy behavior e.g., smoking, drinking alcohol over the limit**	O	O	O	O
15.	**If my friends or siblings have questions about health, I can help them.**	O	O	O	O
16.	**It is easy for me to talk with my parents about health topics.**	O	O	O	O

Scale C. Attitudes toward one's own health and health information

		not at all (1)	little (2)	moderate (3)	strong (4)	very strong (5)
17.	How much in general do you pay attention to your health?	O	O	O	O	O

	To what extent do you agree to the following sentences?	strongly agree (5)	somewhat agree (4)	neither agree or disagree (3)	somewhat disagree (2)	strongly disagree (1)
18.	**I seek advice from others when I am ill.**	O	O	O	O	O
19.	**It is up to me to protect myself from diseases.**	O	O	O	O	O
20.	**I know what I have to do to make myself feel comfortable.**	O	O	O	O	O
21.	**It is important to me to inform myself about health-related topics.**	O	O	O	O	O

Scale D. Health-related knowledge

1. **How often should a young person at your age be physically active?** *Only one answer is correct.*

O Up to two hours of sport per week is sufficient (0)

O Physical education lessons at school are sufficient (0)

O About an hour every day while one's works up a sweat, is sufficient (1)

O Three times a week exercise is sufficient (0)

O Do not know (0)

2. **How does it affect the body if you regularly drink a lot of alcohol?** *Only one answer is correct.*

O Almost all organs are damaged (1)

O Only the liver and brain are damaged (0)

O It depends on how much the body can tolerate (0)

O The lung volume decreases (0)

O Do not know (0)

3. **What are the health effects for young people of consuming cannabis (marijuana, hashish) often?** *Only one answer is correct.*

O Poorer learning and memory skills (1)

O Permanent brain damage (0)

O Hair loss (0)

O Only physical dependence (0)

O Do not know (0)

4. **What is NOT one of the possible effects of smoking?** *Only one answer is correct.*

O Tooth loss (0)

O Skin aging (0)

O Nearsightedness (1)

O Decreased muscular strength (0)

O Do not know (0)

5. **How can small burns be treated?** *Only one answer is correct.*

O Sprinkle flour on the wound (0)

O Cool for 10 minutes under lukewarm water (1)

O Always pierce blisters from burns (0)

O Cool only with sterile saline solution (0)

O Do not know (0)

6. **Which ingredient is contained in the highest amount in a cocoa drink powder with the ingredients listed on the package as follows: sugar, dextrose, low-fat cocoa powder, emulsifier agent (lecithin), salt?** *Only one answer is correct.*

O Salt (0)

O Dextrose (0)

O Sugar (1)

O Cocoa powder (0)

O Do not know (0)

7. **We want to know if you know what your rights are. One of the following sentences is WRONG. Which sentence is that?** ***Only one answer is correct.***

O I can disagree to a major medical surgery, even if my parents have agreed. (0)

O I have to agree before my doctor will inform my parents about my treatment. (0)

O I have the right for the doctor to inform me about the advantages and disadvantages of my treatment. (0)

O I have no rights. (1)

O Do not know (0)

8. **How can HIV/AIDS be transmitted?** *Only one answer is correct*

O Kissing (0)

O Skin contact such as shaking hands (0)

O Insect bites (0)

O None of the given answers is correct (1)

O Do not know (0)
